# Rationally re-designed mutation of NAD-independent l-lactate dehydrogenase: high optical resolution of racemic mandelic acid by the engineered *Escherichia coli*

**DOI:** 10.1186/1475-2859-11-151

**Published:** 2012-11-23

**Authors:** Tianyi Jiang, Chao Gao, Peipei Dou, Cuiqing Ma, Jian Kong, Ping Xu

**Affiliations:** 1 State Key Laboratory of Microbial Technology, Shandong University, Jinan, 250100, China; 2 State Key Laboratory of Microbial Metabolism and Schoolof Life Sciences and Biotechnology, Shanghai Jiao Tong University, Shanghai, 200240, China

**Keywords:** NAD-independent l-lactate dehydrogenase, Site-directed mutagenesis, Optical resolution, d-mandelic acid

## Abstract

**Background:**

NAD-independent l-lactate dehydrogenase (l-iLDH) from *Pseudomonas stutzeri* SDM can potentially be used for the kinetic resolution of small aliphatic 2-hydroxycarboxylic acids. However, this enzyme showed rather low activity towards aromatic 2-hydroxycarboxylic acids.

**Results:**

Val-108 of l-iLDH was changed to Ala by rationally site-directed mutagenesis. The l-iLDH mutant exhibited much higher activity than wide-type l-iLDH towards l-mandelate, an aromatic 2-hydroxycarboxylic acid. Using the engineered *Escherichia coli* expressing the mutant l-iLDH as a biocatalyst, 40 g·L^-1^ of dl-mandelic acid was converted to 20.1 g·L^-1^ of d-mandelic acid (enantiomeric purity higher than 99.5%) and 19.3 g·L^-1^ of benzoylformic acid.

**Conclusions:**

A new biocatalyst with high catalytic efficiency toward an unnatural substrate was constructed by rationally re-design mutagenesis. Two building block intermediates (optically pure d-mandelic acid and benzoylformic acid) were efficiently produced by the one-pot biotransformation system.

## Background

d-Mandelic acid, an aromatic 2-hydroxycarboxylic acid, is a valuable chiral building block for the synthesis of various pharmaceuticals, such as anti-obesity agents, antitumor agents, penicillins, and semisynthetic cephalosporins [1-3]. Chemical processes for mandelic acid production result in the racemic mixture of both stereospecific forms. Several biocatalytic methods, including lipase catalyzed enantioselective esterification [4], oxidoreductase catalyzed enantioselective oxidation, and microbial mediated enantioselective degradation [5-10], have been developed to prepare d-mandelic acid from racemic mandelic acid. Among these routes, oxidative resolution of racemic mandelic acid is much more promising because of its easy manipulation, exclusion of co-substrate addition, and high yield.

**Figure 1:**
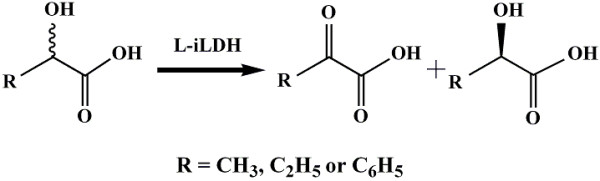
**Scheme for kinetic resolution of 2-hydroxycarboxylic acids.** R is CH_3_: lactic acid [13]; C_2_H_5_: 2-hydroxybutyric acid [14]; C_6_H_5_: mandelic acid.

The NAD-independent l-lactate dehydrogenase (l-iLDH) of *Pseudomonas stutzeri* SDM is located on the cell membrane, and quinine, as its electron acceptor, could be directly regenerated by the membrane electron transport chain [11]. So it may exhibit higher catalytic efficiency than the soluble FMN-dependent α-hydroxyacid dehydrogenases. Previous report showed that it exhibits high catalytic efficiency and enantioselectivity toward small aliphatic 2-hydroxycarboxylic acids such as l-lactate and l-2-hydroxybutanoate [12]. Cells of *P. stutzeri* SDM have been used in the kinetic resolution of lactate and 2-hydroxybutanoate racemic mixtures to produce d-lactate and d-2-hydroxybutanoate [13,14]. Considering the similar structures of lactic acid and mandelic acid, l-iLDH might also be able to catalyze the kinetic resolution of racemic mandelic acid (Figure 1). l-iLDH from *P. stutzeri* SDM was purified, and then it was characterized further [12]. It showed rather low activity towards l-mandelate [12], which restricts its potential application for the kinetic resolution of racemic mandelic acid.

In recent years, rational re-design mutagenesis has emerged as a practical technique for the construction of biocatalysts with high catalytic efficiencies toward unnatural substrates, like the re-design of phenylalanine dehydrogenase [15-19], NAD-dependent l-lactate dehydrogenase [20], and d-amino-acid oxidase [21]. In this study, l-iLDH from *P. stutzeri* SDM was rationally re-designed on the basis of the sequence alignment and active site structure of its homologous enzyme flavocytochrome *b*_2_[22]. The mutant enzyme was expressed in *E. coli*, purified, and characterized. l-Mandelate dehydrogenation activity of the mutant enzyme was successfully enhanced. Whole cells of *E. coli* expressing the mutant l-iLDH were then used to perform the kinetic resolution of racemic mandelic acid.

## Results and discussion

### Rationally re-designed mutation

l-iLDH is a member of the l-α-hydroxyacid-oxidizing flavoprotein family, and is therefore related both structurally and by sequence to a number of other enzymes, including flavocytochrome *b*_2_ from *Saccharomyces cerevisiae*[22]. The X-ray crystal structure of flavocytochrome *b*_2_ has been resolved (PDB ID code 1FCB), and the active site for 2-α-hydroxyacid dehydrogenation has been identified [23]. Six amino acids which interact directly to the substrate have been pinpointed in the crystal structure [24], in which four amino acids are highly conserved in this protein family. However, the other two residues, Ala-198 and Leu-230 in flavocytochrome *b*_2_, which interact with the alkyl group of substrates, are not well conserved (Additional file 1 Figure S1). They are considered important for the substrate specificity of the enzyme [24]. As an aromatic 2-hydroxycarboxylic acid, l-mandelic acid is similar in structure with l-lactic acid except that phenyl group replaces alkyl group (Figure 1). Double mutation of the Ala-198 and Leu-230 to amino acids with smaller side chains (Gly and Ala, respectively) increased the ability of flavocytochrome *b*_2_ to utilize l-mandelate as a substrate [25]. The altered substrate specificity may result from enlargement of the active site space to accommodate the phenyl group [25].

**Figure 2:**
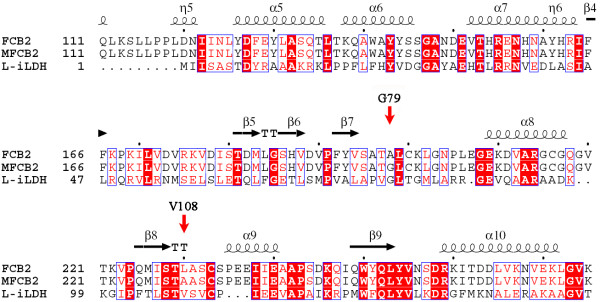
**Sequence comparison of l-iLDH with wild-type and mutant flavocytochrome *b***_**2**_**.**l-iLDH: NAD-independent l-lactate dehydrogenase from *P. stutzeri* SDM [12]; FCB2: flavocytochrome *b*_2_ from *S. cerevisiae*[22]; MFCB2: double-mutant of flavocytochrome *b*_2_ with increased l-mandelate degrading activity [25]. The positions of Gly-79 and Val-108 of l-iLDH are indicated by red arrows. The figure was generated with Clustal X [26] and ESPript [27].

Considering the similarity of active site structures of this protein family, there may also be key residues affecting the substrate specificity of l-iLDH. However, the overall sequence identity of l-iLDH with flavocytochrome *b*_2_ is only 29%, which is relatively low for providing an accurate model of l-iLDH by homology modeling. So sequence alignment was performed to indentify these residues. The result suggests that the corresponding residues of flavocytochrome *b*_2_ Ala-198 and Leu-230 are Gly-79 and Val-108, respectively, in l-iLDH (Figure 2). Gly-79 is the amino acid having smallest side chain and is the same amino acid as in the mutant flavocytochrome *b*_2_ with increasing l-mandelic acid dehydrogenase activity [25]. However, as the Leu-230 in flavocytochrome *b*_2_, the Val-108 of l-iLDH has a larger side chain than Ala, which may narrow the active site space of l-iLDH. Therefore, Val-108 was selected to be mutated to Ala to increase the l-mandelate dehydrogenase activity of l-iLDH.

### Kinetics of V108A l-iLDH

V108A l-iLDH was expressed in *E. coli* C43 (DE3), and it was purified using the protocol reported for the wild-type l-iLDH [12]. The activity of wild-type l-iLDH for l-mandelate was below the detection limit for a reliable measurement with 3-(4, 5-dimethylthiazol-2-yl)-2, 5-diphenyltetrazolium bromide (MTT) as the electron acceptor [12]. However, when the reaction time was extended, the oxidation product benzoylformate could be detected by HPLC, suggesting weak activity of l-iLDH for l-mandelate. The more sensitive electron acceptor 2,6-dichloroindophenol (DCIP) was used to determine the accurate kinetic parameters of the enzyme.

As shown in Table 1, the activity of l-iLDH towards l-mandelate was low, with a *k*_cat_ value of only 8.3 ± 1.3 s^-1^ (compared with a value of 445 ± 44 s^-1^ for l-lactate). The *K*_m_ with l-mandelate as a substrate was 6.8 ± 1.0 mM, which was 38-fold higher than that of l-lactate. The V108A mutant enzyme resulted in a 12-fold increase in the *K*_cat_ value with l-mandelate, and the *K*_m_ was decreased to 1.6 ± 0.1 mM. Therefore, the catalytic efficiency (based on the *k*_cat_/*K*_m_ value) was increased by 50.8-fold for the V108A mutant compared to the wild-type enzyme. The catalytic efficiency of the mutant enzyme with l-lactate had a nearly 10 times lost. However, the value was still 3.8 folds higher than with l-mandelate, which differed from the case of *S. cerevisiae* flavocytochrome *b*_2_, in which the optimum substrate was altered from l-lactate to l-mandelate by the double mutation [25]. No reverse action activity or activity with d-mandelate of the mutant l-iLDH was detected. d-Mandelate competitively inhibited V108A l-iLDH activity towards l-mandelate. The *K*_i_ value was estimated to be 5.5 ± 0.5 mM (Additional file 2 Figure S2). Effect of the mutation on stability of the mutated enzyme in comparison with the wild-type enzyme was also investigated. Within the selected range, no remarkable change of the enzyme stability as function of temperature and pH was observed (Additional file 3 Figure S3).

**Table 1:** **Kinetics of wild-type (WT) and V108Al-iLDH**

**Substrate**	**WTl-iLDH**			**V108Al-iLDH**		
	***K***_**cat**_**(s**^**-1**^**)**	***K***_**m**_**(mM)**	***K***_**cat**_**/*K***_**m**_**(mM**^**-1**^**s**^**-1**^**)**	***K***_**cat**_**(s**^**-1**^**)**	***K***_**m**_**(mM)**	***K***_**cat**_**/*K***_**m**_**(mM**^**-1**^**s**^**-1**^**)**
l-Lactate	445 ± 44	0.18 ± 0.01	2472	185 ± 36	0.8 ± 0.1	231
l-Mandelate	8.3 ± 1.3	6.8 ± 1.0	1.2	97 ± 13	1.6 ± 0.1	61

All experiments were carried out at 30°C in 1 mL of 50 mM Tris–HCl (pH 7.5), with DCIP (0.0625 mM) as the electron acceptor. Values for *k*_cat_ are expressed as electrons transferred per second per molecule of enzyme. *K*_m_ values are expressed in terms of mM of substrate.

### Feasibility in co-production of d-mandelic acid and benzoylformic acid

**Figure 3:**
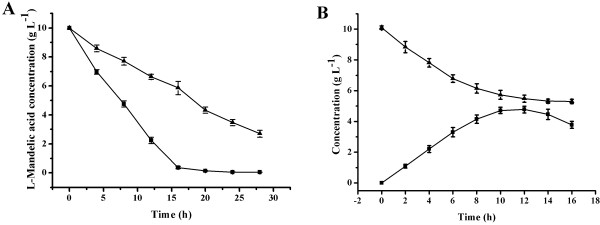
**Identifying the feasibility of co-production of d-mandelic acid and benzoylformic acid by *E. coli *expressing V108A l-iLDH.** (**A**) Comparison of capacities for degrading l-mandelic acid of *E. coli* strains expressing wild-type or V108A l-iLDH. (▴) The catalytic process of the *E. coli* strain expressing wild-type l-iLDH; (▪) The catalytic process of the *E. coli* strain expressing V108A l-iLDH. (**B**) The catalytic process of the *E. coli* strain expressing V108A l-iLDH with racemic mandelic acid as the substrate. (▴) Concentration of racemic mandelic acid; (▪) Concentration of benzoylformic acid. Values are the mean ± SD of 3 separate determinations.

Since the mutant l-iLDH was shown to oxidize l-mandelate to benzoylformate, the feasibility of kinetic resolution of dl-mandelic acid by the enzyme was further studied. *E. coli*, a non-native microbe for the degradation of mandelic acid, was selected as a suitable host for the kinetic resolution of dl-mandelic acid. To verify that the engineered *E. coli* strains exhibited mandelic acid oxidation activity, the capacity for l-mandelic acid oxidation of the 2 *E. coli* strains expressing wild type or V108A l-iLDH was detected and compared. As shown in Figure 3A, by using 12.5 g dry cell weight (DCW) L^-1^ of *E. coli* expressing V108A l-iLDH as the biocatalyst and 10 g·L^-1^ of l-mandelic acid as the substrate, l-mandelic acid in the reaction system was degraded completely within 20 h. By contrast, 2.7 g·L^-1^ of l-mandelic acid remained after 28 h of reaction with the same reaction system by using the cells expressing wild-type enzyme. Furthermore, when using racemic mandelic acid as the substrate, about half of total mandelic acid could not be degraded (Figure 3B), which was determined to be the d-enantiomer (Additional file 4 Figure S4). The inhibition effect of d-mandelic acid may have on the rate of reaction was further investigated. Different concentrations of d-mandelic acid (from 0 g·L^-1^ to 30 g·L^-1^) were added to reaction system containing 10 g·L^-1^l-mandelic acid, and biocatalyst activity was evaluated after 4 h of reaction. The result showed that d-mandelic acid did not affect the catalytic efficiency remarkably (Additional file 5 Figure S5). These results suggest that the whole-cell biocatalyst of *E. coli* expressing V108A l-iLDH has the potential to produce highly pure d-mandelic acid and benzoylformic acid from racemic mandelic acid.

### Optimization of biocatalysis conditions

To increase the efficiency of whole-cell biocatalysis in the kinetic resolution of dl-mandelic acid, the bioconversion conditions were optimized. Benzoylformic acid could be degraded by microbial cells as other 2-keto-acids (Figure 3B and Additional file 6 Figure S6). The degradation decreased the bioconversion ratio and produced some by-products. EDTA was added at a concentration of 20 mM in the reaction system, which removed bivalent ions necessary for 2-keto-acid decarboxylase-catalyzed reactions [28] and then prevents the degradation of benzoylformic acid (Additional file 6 Figure S6).

**Figure 4:**
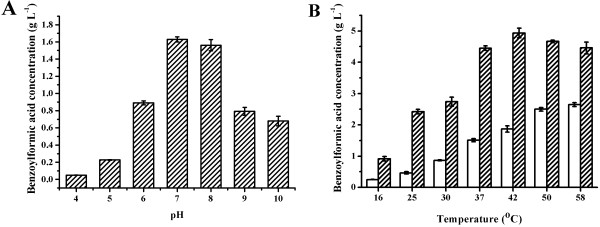
**Optimization of pH and temperature for biocatalysis.** (**A**) Optimization of pH. (**B**) Optimization of temperature. Unshaded bars represent the production of benzoylformic acid in 4 h. Shaded bars represent the production of benzoylformic acid in 10 h. Values are the mean ± SD of 3 separate determinations.

Since pH and temperature are parameters that often limit enzyme activity and stability in technical applications, studies addressing the effects of temperature and pH on whole-cell catalysis were performed. The optimal pH was found to be 7.0 after adjusting the pH of the reaction system from 4.0 to 10.0 (Figure 4A). The effect of the reaction temperature was examined in the range of 16°C to 58°C after 4 h and 10 h of reaction. As shown in Figure 4B, with increasing temperature, higher activity of the biocatalyst was observed over the short term, as shown by the benzoylformic acid production after 4 h of reaction. However, the activity decreased more rapidly at higher temperatures, as shown by the benzoylformic acid production after 10 h of reaction. The optimal reaction temperature was chosen to be 42°C, which optimized enzyme activity and stability. Either the lower activity at lower temperature or the lower stability at higher temperature may cause a decrease of overall l-mandelic acid degrading capacity of the biocatalyst, which will then decrease the production capacity of d-mandelic acid.

The optimal concentration of dl-mandelic acid for the biotransformation was then determined (Table 2). When the biocatalyst was prepared from 25 g (DCW) L^-1^ of *E. coli*, the biotransformation efficiency only decreased slightly as the dl-mandelic acid concentration increased. A higher substrate concentration will result in higher concentrations of products and simplifies the downstream process. The reaction with 40 g·L^-1^ of dl-mandelic acid as the substrate exhibited relatively high concentrations of benzoylformic acid and d-mandelic acid with high optical purity.

**Table 2:** **Effect ofdl-mandelic acid concentration on the biotransformation**

	**dl-Mandelic acid concentration (g·L**^**-1**^**)**				
	**10**	**20**	**30**	**40**	**50**
Reaction time (h)	8	18	30	42	54
Benzoylformic acid concentration (g·L^-1^)	4.8	9.8	14.3	19.1	15.6
Biotransformation efficiency (g·L^-1^·h^-1^)	0.62	0.55	0.50	0.47	0.46
Residual l-mandelic acid (g·L^-1^)	< 0.005				9.6
Residual d-mandelic acid (g·L^-1^)	5.0	10.1	15.1	20.1	25.2
Enantiomeric excess (%)	> 99.5				45.0

Values are the mean of 3 separate determinations.

**Figure 5:**
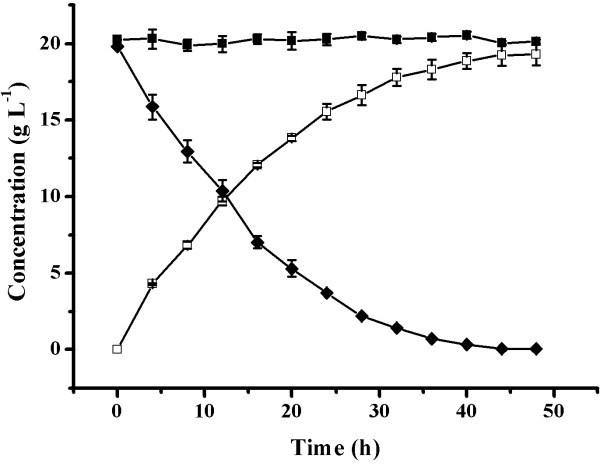
**Time course of the kinetic resolution of racemic mandelic acid by whole cells of *E. coli *expressing V108A l-iLDH.** (♦) l-mandelic acid; **(**▪) d-mandelic acid; (□) benzoylformic acid. Accurate concentrations of benzoylformic acid and mandelic acid in the reaction mixture were analyzed by HPLC at the indicated times. Values are the mean ± SD of 3 separate determinations.

**Figure 6:**
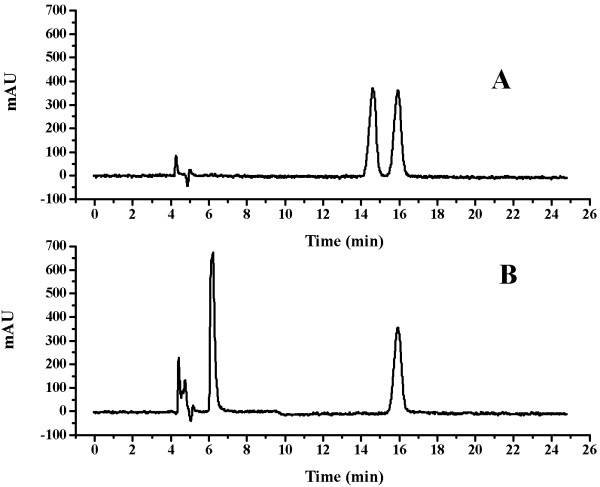
**Analysis of optical purity of mandelic acid by HPLC.** (**A**) Sample at the beginning of the biotransformation. **(B**) Sample at the end of the biotransformation.

As shown in Figure 5, under optimal conditions, a concentration of 20.1 g·L^-1^d-mandelic acid and 19.3 g·L^-1^ benzoylformic acid was obtained from 40 g·L^-1^ of dl-mandelic acid after 42 h in a batch bioconversion. The biotransformation produced high enantiomeric excess (> 99.5%) of d-mandelic acid (Figure 6) at high concentrations of d-mandelic acid and benzoylformic acid. Kinetic resolution of dl-mandelic acid also could be performed by other biocatalysis processes. However, the processes using lipase or nitrilase resulted in rather low d-mandelic acid concentrations [1-4,29-31]. Enantioselective oxidation of the l-enantiomer from racemic mandelic acid to prepare d-enantiomer is an attractive procedure because it uses inexpensive starting material and has high product yield. Although *Pseudomonas* sp. [5-7] and *Alcaligenes* sp. [8-10] have been previously used to produce d-mandelic acid by this route, the simple active conversion system in this study is a promising alternative because *E. coli* is much easier to operate, the d-mandelic acid degrading activity is totally excluded, and resulting in high yields of the 2 building-block intermediates (d-mandelic acid and benzoylformic acid) by a one-pot biotransformation method.

## Conclusions

In this work, l-iLDH was rationally re-designed on the basis of sequence alignment and the active site structure of a homologous enzyme; a new biocatalyst with high catalytic efficiency toward an unnatural substrate was successfully constructed. A one-pot biotransformation system producing 2 building block intermediates was established using the biocatalyst. Under optimal conditions, a concentration of 20.1 g·L^-1^ of d-mandelic acid with high enantiomeric excess (> 99.5%) and 19.3 g·L^-1^ of benzoylformic acid was obtained from 40 g·L^-1^ of dl-mandelic acid.

## Materials and methods

### Enzymes and chemicals

Restriction enzymes were purchased from TaKaRa Bio Inc (China). FastPfu DNA polymerase and T_4_ DNA ligase were purchased from Transgen Biotech (China) and MBI (USA), respectively. d-mandelic acid, l-mandelic acid, racemic mandelic acid, benzoylformic acid, and 2,6-dichloroindophenol (DCIP) were all purchased from Sigma-Aldrich (USA). Isopropyl-β-D-1-thiogalactopyranoside (IPTG), dithiothreitol (DTT), and phenylmethanesulfonyl fluoride (PMSF) were obtained from Merck (Germany). All other chemicals were of reagent grade.

### Site-directed mutagenesis and enzyme expression

To construct the mutant enzyme with valine-108 changed to alanine (V108A l-iLDH), site-directed mutagenesis was performed using the MutanBEST Kit (TaKaRa). The *lldD* gene coding for the wild-type l-iLDH was subcloned into the pMD^TM^18-T vector (TaKaRa) to construct the pMD^TM^18-T-*lldD* vector. The mutation was introduced using the following primers: 5^′^TTCACCCTTTCCACCGCGTCGGTCT3^′^ and 5^′^GGGAATCCCTTTCTTGTCTGCCGC3^′^ to amplify the entire sequence of pMD^TM^18-T-*lldD*. The linear PCR products were then cyclized using the ligase from the MutanBEST Kit. The mutant *lldD* gene (confirmed by automated DNA sequencing) was subcloned into the *Hind*III and *Xho*I restriction sites of the pETDuet-1 expression vector with a T7 promoter. Plasmid purification, DNA manipulation, and transformation were performed by the standard methods described by Sambrook et al. [32]. *E. coli* DH5α and C43 (DE3) were used for general cloning and expression procedures, respectively. Lysogenic broth (LB) medium was used for *E. coli* cultivations. Ampicillin was used at a concentration of 100 μg· mL^-1^.

The expression and purification procedure of wild-type l-iLDH from recombinant *E. coli* has been described previously [12]. The same procedure was used to express and purify the V108A mutant of l-iLDH. The purified enzymes were concentrated by ultrafiltration, desalted with Sephadex G-25, and then stored in 100 mM sodium phosphate buffer (pH 8.0, containing 0.1% Triton X-100) at −20°C. The expressed and purified enzyme was confirmed by sodium dodecyl sulfate polyacrylamide gel electrophoresis (SDS-PAGE).

### Biochemical assays

The activities of wild-type and mutant l-iLDH were determined at 30°C in 1 mL of 50 mM Tris–HCl, pH 7.5, 0.0625 mM DCIP, and the enzyme. Protein amounts of wild-type enzyme and V108A l-iLDH mutant used to assay the kinetic parameters towards l-lactate were 0.05 μg and 0.2 μg, repectively; 4.0 μg and 1.0 μg proteins of wild-type and V108A l-iLDH were used to assay the kinetic parameters towards l-mandelate, respectively. The reaction was started by the addition of l-lactate or l-mandelate, and the rates of DCIP reduction were determined by measuring the absorbance change at 600 nm [33]. To study effects of temperature and pH on enzyme stability, the enzyme was incubated at different temperature for 0.5 h or at different pH for 2 h, and then assayed with 1.25 mM l-lactate as substrate. MTT was used at a concentration of 0.2 mM instead of 0.0625 mM DCIP as electron acceptor in the assay of pH stability, since the molar extinction coefficient of DCIP changes with different pH. The rate of MTT reduction was determined by measuring changes in absorbance at 578 nm [34]. One unit of l-iLDH activity was defined as the amount reducing 1.0 μmol of electron acceptor per minute under the test conditions. Protein concentration was determined by the Lowry method with BSA as a standard [35].

### Biocatalyst preparation

Recombinant *E. coli* C43 (DE3) cells were grown at 37°C on a rotary shaker (180 rpm) in LB medium containing ampicillin (100 μg·mL^-1^) to an OD_620_ of 0.6. Expression of the recombinant gene was induced by adding 1 mM IPTG at the same temperature for 6 h. After induction, the cells were harvested by centrifugation at 6,000 × *g* for 10 min at 4°C and then washed twice with 0.85% NaCl. The whole cells resuspended in ddH_2_O were used as the biocatalyst for the kinetic resolution of dl-mandelic acid.

### Optimization of biotransformation conditions

To optimize the biotransformation conditions, 20-mL samples of the reaction mixture in a 100-mL flask were used. Ethylenediaminetetraacetic acid (EDTA) was added to a concentration of 20 mM in the reaction system. The biocatalysts were prepared from 12.5 g (DCW) L^-1^ of *E. coli* C43 (DE3) expressing the V108A mutant l-iLDH for the optimization of pH and temperature. The pH was adjusted from 4.0 to 10.0. The temperatures ranges were from 16°C to 58°C. The activity of whole-cell biocatalyst was judged by the concentration of benzoylformic acid produced. The dl-mandelic acid concentrations were 10.0–50.0 g·L^-1^, and the biotransformation was performed at 42°C at pH 7.0 for 12–54 h with 25 g (DCW) L^-1^ of biocatalyst.

### Analytical methods

Accurate concentrations of mandelic acid and benzoylformic acid were analyzed by high-performance liquid chromatography (HPLC, Agilent 1100 series, USA) using an Aminex HPX-87H column (Bio-Rad, USA) and the eluent using 10 mM H_2_SO_4_ solution at a flow rate of 0.4 mL·min^-1^. Samples from reaction systems during biocatalysis were centrifuged at 140,000 × *g* for 5 min to remove cells, and the supernatant was filtered by 0.22 μm pore size membrane filter for HPLC analysis.

Stereoselective assays of d-mandelic acid and l-mandelic acid were performed by HPLC analysis using a chiral column (DAICEL CHIRALCEL OJ-RH, Japan) and a tunable UV detector at 205 nm. The mobile phase consisted of 90% H_2_O (with 0.1% acetic acid added) and 10% acetonitrile (v/v) pumped at 0.4 mL·min^-1^ (15°C). Samples were prepared by the same procedure as using Aminex HPX-87H column. The optical purity of d-mandelate was expressed as enantiomeric excess (ee value), which was defined as the ratio of $\frac{\left(D-mandelic\;acid\right)-\left(L-mandelic\;acid\right)}{\left(D-mandelic\;acid\right)+\left(L-mandelic\;acid\right)}x100\%$.

## Competing interests

The authors declare that they have no competing interests.

## Authors’ contributions

PX and CM designed experiments. TJ, CG and PD performed experiments. CM and JK contributed reagents and materials. TJ and CG analyzed data. TJ, PX and CM wrote the manuscript. All authors have read and approved the final manuscript.

## Supplementary Material

Additional file 1 **Figure S1.The active site structure of flavocytochrome*b***_**2**_**.** The figure is generated according to the molecular structure of flavocytochrome *b*_2_ at 2.4 Å resolution (PDB code 1FCB) [23] with PyMOL (The PyMOL Molecular Graphics System, Version 0.99rc6, Schrödinger, LLC). The pyruvate ligand, as oxidation product of l-lactate, is shown in orange. The residues interact directly to the substrate are shown in blue. The residue labels in parentheses are the corresponding residues of l-iLDH, which are identified by sequence alignment. Click here for file

Additional file 2 **Figure S2.Inhibition of V108AL-iLDH byD-mandelate.** Purified V108A l-iLDH (1 μg) was incubated in the reaction mixture contained 0.0625 mM DCIP and 50 mM Tris–HCl (pH 7.5) at 30°C. The reaction was started with different l-mandelate concentrations at variable d-mandelate concentrations. ▪, no d-mandelate; ·, 6.25 mM d-mandelate; ▴, 12.5 mM d-mandelate; ▾, 25 mM d-mandelate; ◂, 37.5 mM d-mandelate. The patterns of double-reciprocal plots indicate a competitive inhibition. The *K*_i_ value was estimated to be 5.5 ± 0.5 mM. Click here for file

Additional file 3 **Figure S3.The stability of wild-type and V108AL-iLDH as function of temperature and pH.** (A) The effect of temperature on the enzyme stability. The enzyme was incubated at different temperature ranging from 16°C to 58°C for 0.5 h and then assayed. The enzyme activity without treatment (store at 4°C) was defined as 100%. ▪, wild-type L-iLDH; ·, V108A L-iLDH. (B) The effect of pH on the enzyme stability. The enzyme was incubated at different pH ranging from 3.0 to 11.0 for 2 h and then assayed. The buffers were: 0.2 M Na_2_HPO_4_-0.1 M citric acid buffer for pH 3.0-8.0; 50 mM Glycine-NaOH buffer for pH 8.0-12.0. The enzyme activities of wild-type and V108A l-iLDH without pH treatment (stored in 100 mM sodium phosphate buffer, pH 8.0) was defined as 100% severally. MTT was used at a concentration of 0.2 mM instead of 0.0625 mM DCIP as electron acceptor in the assay of pH stability, for the molar extinction coefficient of DCIP changes with different pH. ▪, wild-type L-iLDH; ·, V108A L-iLDH. Values are the mean ± SD of 3 separate determinations. Click here for file

Additional file 4 **Figure S4.HPLC analysis of the chiral products of the reaction catalyzed by V108AL-iLDH.** (A) Authentic d-mandelic acid; (B) authentic l-mandelic acid; (C) reaction mixture at the beginning of the reaction (solid line), after 4 h (short dot line), and after 10 h (dash dot line). The biotransformation was carried out using 12.5 g (DCW) L^-1^ of *E. coli* expressing V108A l-iLDH as the biocatalyst and 10 g·L^-1^dl-mandelic acid as the substrate. The analytical methods are described in the “Materials and methods”. Click here for file

Additional file 5 **Figure S5.The inhibition effect ofD-mandelic acid on the whole-cell biocatalyst activity.** Different concentrations of d-mandelic acid (from 0 g·L^-1^ to 30 g·L^-1^) were added to reaction systems containing 10 g·L^-1^l-mandelic acid. The activity of whole-cell biocatalyst was judged by the concentration of benzoylformic acid produced within 4 h of reaction. Values are the mean ± SD of 3 separate determinations. Click here for file

Additional file 6 **Figure S6.HPLC analysis of the products from the reaction catalyzed by V108AL-iLDH.** (A) Authentic mandelic acid; (B) authentic benzoylformic acid; (C) reaction mixture after 8 h of reaction without adding EDTA; (D) reaction mixture after 8 h of reaction with 20 mM EDTA added. The biotransformation was carried out using 25 g (DCW) L^-1^ of *E. coli* expressing V108A l-iLDH as the biocatalyst and 10 g·L^-1^dl-mandelic acid as the substrate. The analytical methods are described in the “Materials and methods.” Click here for file
