# Supplementary material for: Rationally re-designed mutation of NAD-independent l-lactate dehydrogenase: high optical resolution of racemic mandelic acid by the engineered Escherichia coli
Source: Microb Cell Fact. 2012 Nov 23;11:151. doi: 10.1186/1475-2859-11-151 (PMC3526519; doi:10.1186/1475-2859-11-151)
Supplement: Additional file 1 — Figure S1.The active site structure of flavocytochromeb2. The figure is generated according to the molecular structure of flavocytochrome b2 at 2.4 Å resolution (PDB code 1FCB) [23] with PyMOL (The PyMOL Molecular Graphics System, Version 0.99rc6, Schrödinger, LLC). The pyruvate ligand, as oxidation product of l-lactate, is shown in orange. The residues interact directly to the substrate are shown in blue. The residue labels in parentheses are the corresponding residues of l-iLDH, which are identified by sequence alignment. [file 1475-2859-11-151-S1.pdf]

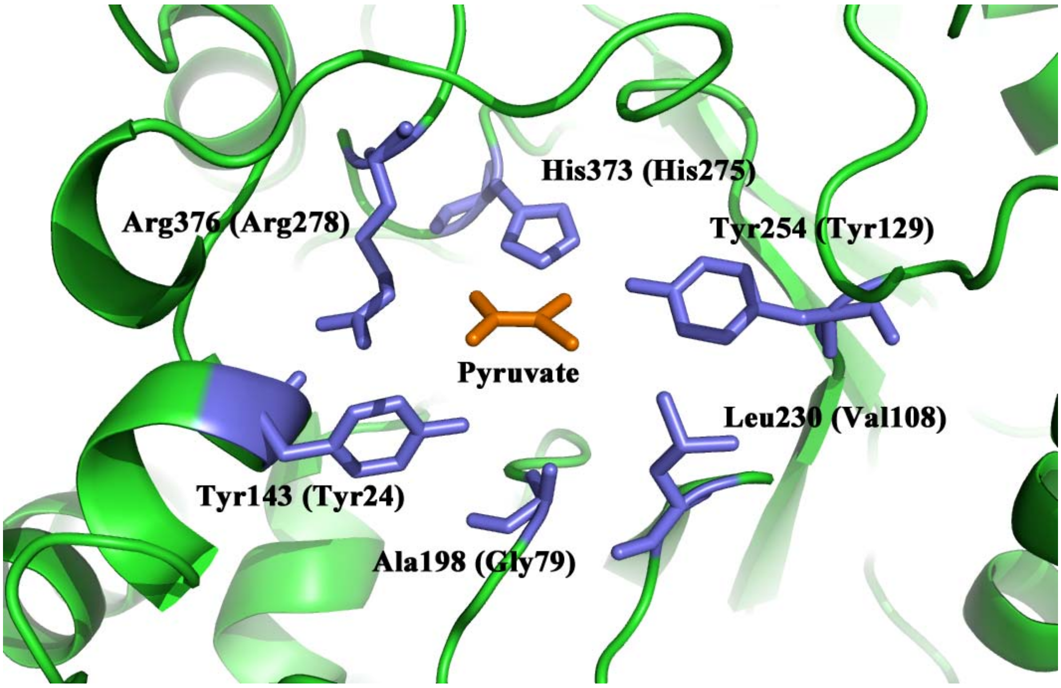

Additional Figure 1. The active site structure of flavocytochrome *b*$_2$. The figure is generated according to the molecular structure of flavocytochrome *b*$_2$ at 2.4 Å resolution (PDB code 1FCB) [23] with PyMOL (The PyMOL Molecular Graphics System, Version 0.99rc6, Schrödinger, LLC). The pyruvate ligand, as oxidation product of L-lactate, is shown in orange. The residues interact directly to the substrate are shown in blue. The residue labels in parentheses are the corresponding residues of L-iLDH, which are identified by sequence alignment.
