# Supplementary material for: Rationally re-designed mutation of NAD-independent l-lactate dehydrogenase: high optical resolution of racemic mandelic acid by the engineered Escherichia coli
Source: Microb Cell Fact. 2012 Nov 23;11:151. doi: 10.1186/1475-2859-11-151 (PMC3526519; doi:10.1186/1475-2859-11-151)
Supplement: Additional file 2 — Figure S2.Inhibition of V108AL-iLDH byD-mandelate. Purified V108A l-iLDH (1 μg) was incubated in the reaction mixture contained 0.0625 mM DCIP and 50 mM Tris–HCl (pH 7.5) at 30°C. The reaction was started with different l-mandelate concentrations at variable d-mandelate concentrations. ▪, no d-mandelate; ·, 6.25 mM d-mandelate; ▴, 12.5 mM d-mandelate; ▾, 25 mM d-mandelate; ◂, 37.5 mM d-mandelate. The patterns of double-reciprocal plots indicate a competitive inhibition. The Ki value was estimated to be 5.5 ± 0.5 mM. [file 1475-2859-11-151-S2.pdf]

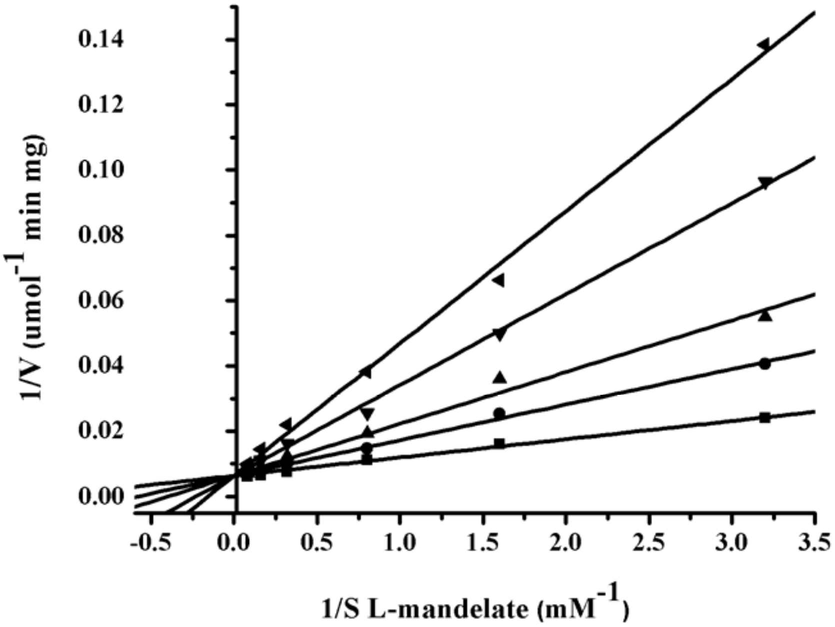

Additional Figure 2. Inhibition of V108A L-iLDH by D-mandelate. Purified V108A L-iLDH (1 μg) was incubated in the reaction mixture contained 0.0625 mM DCIP and 50 mM Tris–HCl (pH 7.5) at 30°C. The reaction was started with different L-mandelate concentrations at variable D-mandelate concentrations. ■, no D-mandelate; ●, 6.25 mM D-mandelate; ▲, 12.5 mM D-mandelate; ▼, 25 mM D-mandelate; ◄, 37.5 mM D-mandelate. The patterns of double-reciprocal plots indicate a competitive inhibition. The K_i value was estimated to be 5.5 ± 0.5 mM.
