# Supplementary material for: Rationally re-designed mutation of NAD-independent l-lactate dehydrogenase: high optical resolution of racemic mandelic acid by the engineered Escherichia coli
Source: Microb Cell Fact. 2012 Nov 23;11:151. doi: 10.1186/1475-2859-11-151 (PMC3526519; doi:10.1186/1475-2859-11-151)
Supplement: Additional file 3 — Figure S3.The stability of wild-type and V108AL-iLDH as function of temperature and pH. (A) The effect of temperature on the enzyme stability. The enzyme was incubated at different temperature ranging from 16°C to 58°C for 0.5 h and then assayed. The enzyme activity without treatment (store at 4°C) was defined as 100%. ▪, wild-type L-iLDH; ·, V108A L-iLDH. (B) The effect of pH on the enzyme stability. The enzyme was incubated at different pH ranging from 3.0 to 11.0 for 2 h and then assayed. The buffers were: 0.2 M Na2HPO4-0.1 M citric acid buffer for pH 3.0-8.0; 50 mM Glycine-NaOH buffer for pH 8.0-12.0. The enzyme activities of wild-type and V108A l-iLDH without pH treatment (stored in 100 mM sodium phosphate buffer, pH 8.0) was defined as 100% severally. MTT was used at a concentration of 0.2 mM instead of 0.0625 mM DCIP as electron acceptor in the assay of pH stability, for the molar extinction coefficient of DCIP changes with different pH. ▪, wild-type L-iLDH; ·, V108A L-iLDH. Values are the mean ± SD of 3 separate determinations. [file 1475-2859-11-151-S3.pdf]

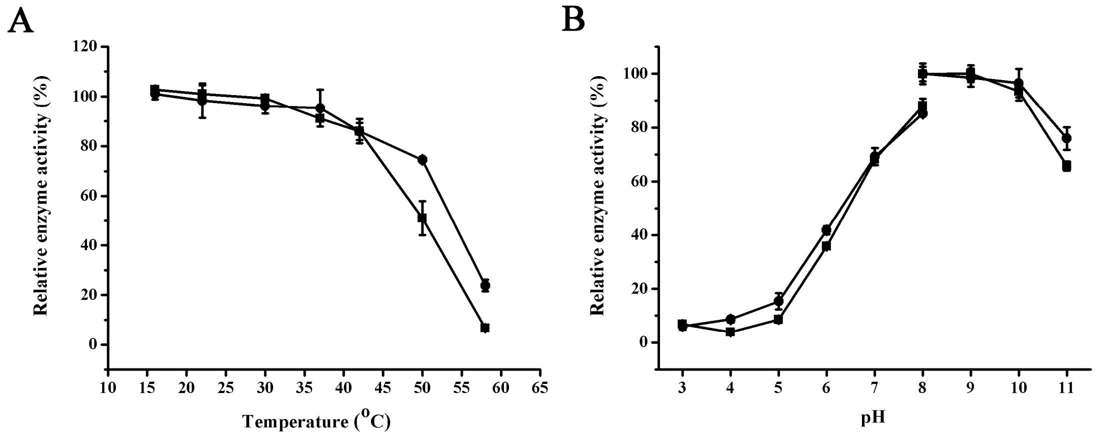

Additional Figure 3. The stability of wild-type and V108A L-iLDH as function of temperature and pH. (A) The effect of temperature on the enzyme stability. The enzyme was incubated at different temperature ranging from 16ºC to 58ºC for 0.5 h and then assayed. The enzyme activity without treatment (store at 4ºC) was defined as 100%. ■, wild-type l-iLDH; ●, V108A l-iLDH. (B) The effect of pH on the enzyme stability. The enzyme was incubated at different pH ranging from 3.0 to 11.0 for 2 h and then assayed. The buffers were: 0.2 M Na_2HPO_4-0.1 M citric acid buffer for pH 3.0-8.0; 50 mM Glycine-NaOH buffer for pH 8.0-12.0. The enzyme activities of wild-type and V108A L-iLDH without pH treatment (stored in 100 mM sodium phosphate buffer, pH 8.0) was defined as 100% severally. MTT was used at a concentration of 0.2 mM instead of 0.0625 mM DCIP as electron acceptor in the assay of pH stability, for the molar extinction coefficient of DCIP changes with different pH. ■, wild-type l-iLDH; ●, V108A l-iLDH. Values are the mean ± SD of 3 separate determinations.
