# Supplementary material for: Rationally re-designed mutation of NAD-independent l-lactate dehydrogenase: high optical resolution of racemic mandelic acid by the engineered Escherichia coli
Source: Microb Cell Fact. 2012 Nov 23;11:151. doi: 10.1186/1475-2859-11-151 (PMC3526519; doi:10.1186/1475-2859-11-151)
Supplement: Additional file 4 — Figure S4.HPLC analysis of the chiral products of the reaction catalyzed by V108AL-iLDH. (A) Authentic d-mandelic acid; (B) authentic l-mandelic acid; (C) reaction mixture at the beginning of the reaction (solid line), after 4 h (short dot line), and after 10 h (dash dot line). The biotransformation was carried out using 12.5 g (DCW) L-1 of E. coli expressing V108A l-iLDH as the biocatalyst and 10 g·L-1dl-mandelic acid as the substrate. The analytical methods are described in the “Materials and methods”. [file 1475-2859-11-151-S4.pdf]

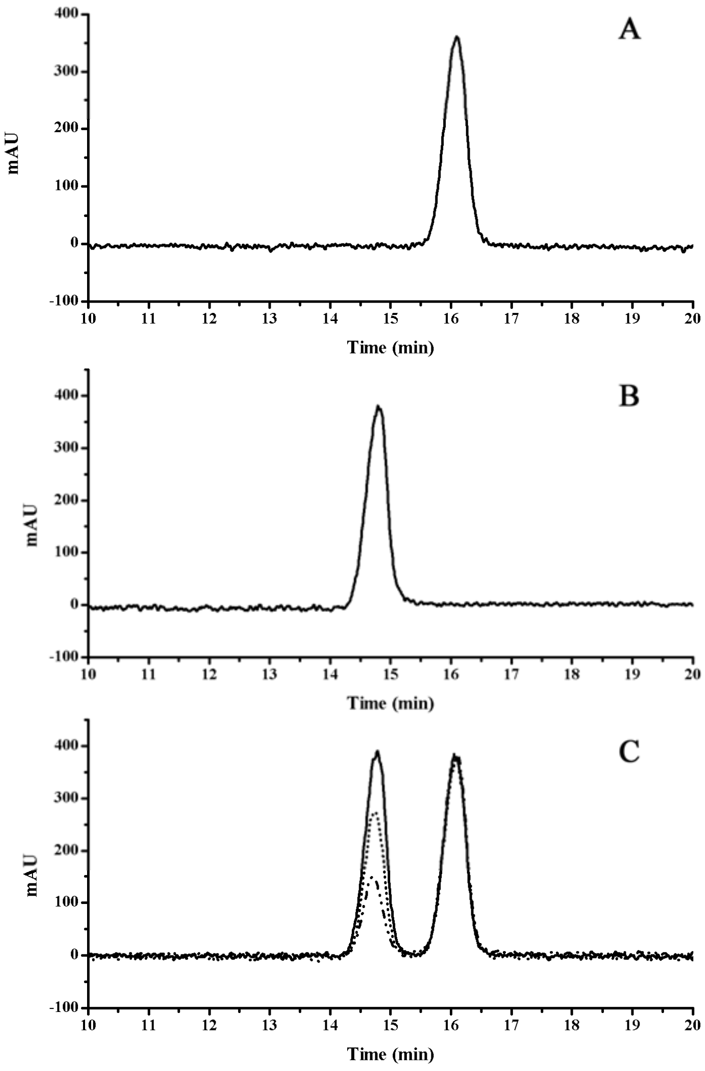

Additional Figure 4. HPLC analysis of the chiral products of the reaction catalyzed by V108A L-iLDH. (A) Authentic D-mandelic acid; (B) authentic L-mandelic acid; (C) reaction mixture at the beginning of the reaction (solid line), after 4 h (short dot line), and after 10 h (dash dot line). The biotransformation was carried out using 12.5 g (DCW) L^{-1} of *E. coli* expressing V108A L-iLDH as the biocatalyst and 10 g·L^{-1} DL-mandelic acid as the substrate. The analytical methods are described in the “Materials and methods”.
