# Supplementary material for: Rationally re-designed mutation of NAD-independent l-lactate dehydrogenase: high optical resolution of racemic mandelic acid by the engineered Escherichia coli
Source: Microb Cell Fact. 2012 Nov 23;11:151. doi: 10.1186/1475-2859-11-151 (PMC3526519; doi:10.1186/1475-2859-11-151)
Supplement: Additional file 5 — Figure S5.The inhibition effect ofD-mandelic acid on the whole-cell biocatalyst activity. Different concentrations of d-mandelic acid (from 0 g·L-1 to 30 g·L-1) were added to reaction systems containing 10 g·L-1l-mandelic acid. The activity of whole-cell biocatalyst was judged by the concentration of benzoylformic acid produced within 4 h of reaction. Values are the mean ± SD of 3 separate determinations. [file 1475-2859-11-151-S5.pdf]

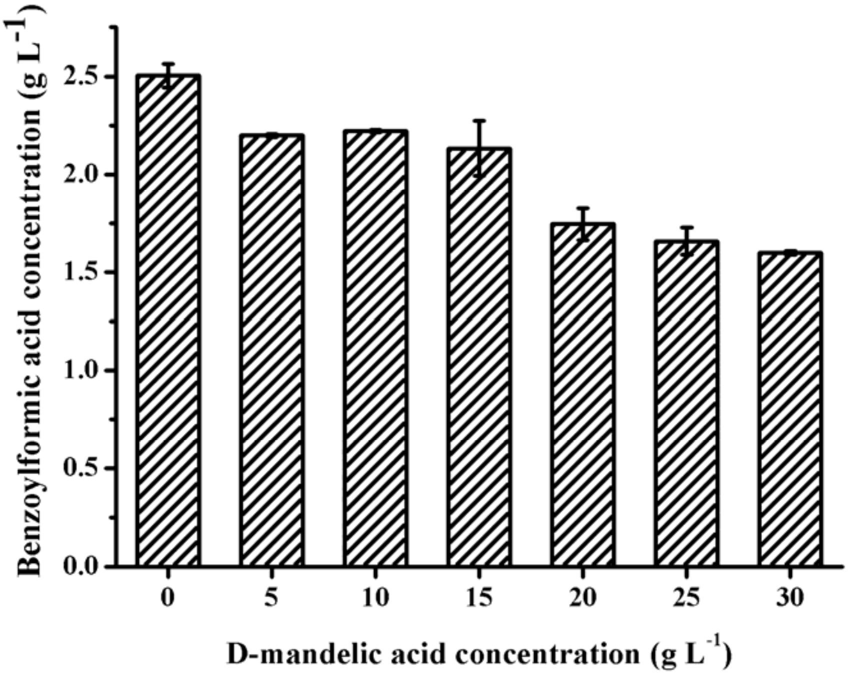

Additional Figure 5. The inhibition effect of D-mandelic acid on the whole-cell biocatalyst activity. Different concentrations of D-mandelic acid (from 0 g·L^{-1} to 30 g·L^{-1}) were added to reaction systems containing 10 g·L^{-1} L-mandelic acid. The activity of whole-cell biocatalyst was judged by the concentration of benzoylformic acid produced within 4 h of reaction. Values are the mean ± SD of 3 separate determinations.
