# Supplementary material for: Rationally re-designed mutation of NAD-independent l-lactate dehydrogenase: high optical resolution of racemic mandelic acid by the engineered Escherichia coli
Source: Microb Cell Fact. 2012 Nov 23;11:151. doi: 10.1186/1475-2859-11-151 (PMC3526519; doi:10.1186/1475-2859-11-151)
Supplement: Additional file 6 — Figure S6.HPLC analysis of the products from the reaction catalyzed by V108AL-iLDH. (A) Authentic mandelic acid; (B) authentic benzoylformic acid; (C) reaction mixture after 8 h of reaction without adding EDTA; (D) reaction mixture after 8 h of reaction with 20 mM EDTA added. The biotransformation was carried out using 25 g (DCW) L-1 of E. coli expressing V108A l-iLDH as the biocatalyst and 10 g·L-1dl-mandelic acid as the substrate. The analytical methods are described in the “Materials and methods.” [file 1475-2859-11-151-S6.pdf]

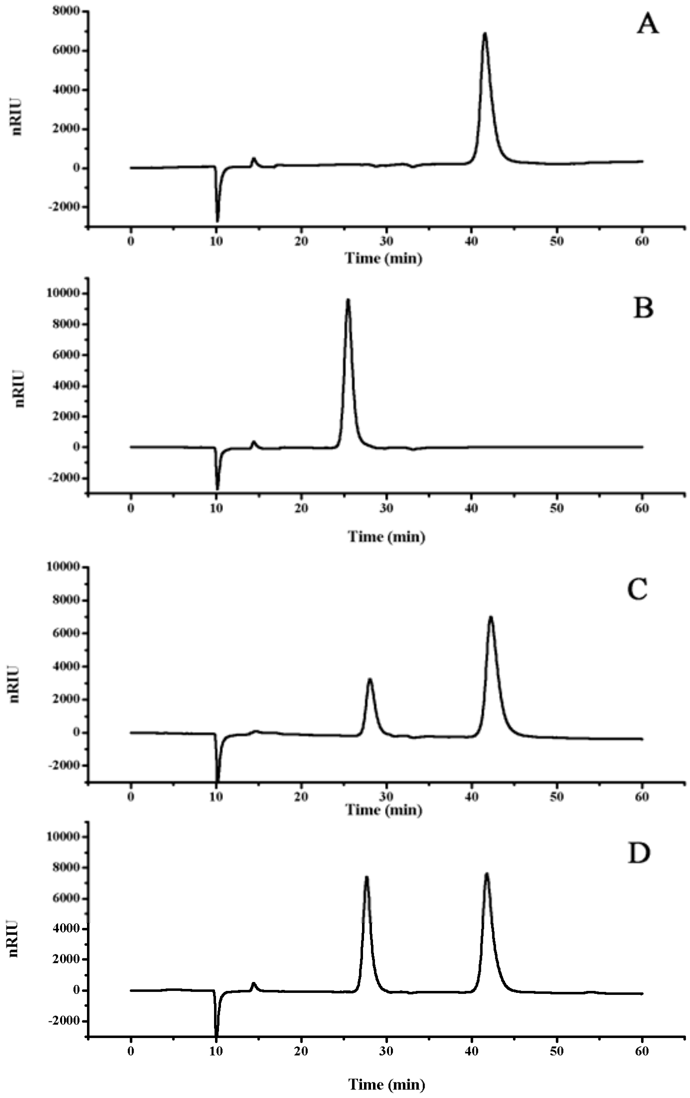

Additional Figure 6. HPLC analysis of the products from the reaction catalyzed by V108A L-iLDH. (A) Authentic mandelic acid; (B) authentic benzoylformic acid; (C) reaction mixture after 8 h of reaction without adding EDTA; (D) reaction mixture after 8 h of reaction with 20 mM EDTA added. The biotransformation was carried out using 25 g (DCW) L^{-1} of *E. coli* expressing V108A L-iLDH as the biocatalyst and 10 g·L^{-1} DL-mandelic acid as the substrate. The analytical methods are described in the "Materials and methods."
